# Are there differences in treatment effects between labial and lingual fixed orthodontic appliances? A systematic review and meta-analysis

**DOI:** 10.1186/s12903-017-0424-z

**Published:** 2017-11-22

**Authors:** Fadi Ata-Ali, Teresa Cobo, Felix De Carlos, Juan Cobo, Javier Ata-Ali

**Affiliations:** 1 Department of Surgery and Medical-Surgical Specialities, Area of Orthodontics, University Medical and Dental School, University of Oviedo, Instituto Asturiano de Odontología, Oviedo, Spain; 2 Department of Dentistry, European University of Valencia, Valencia, Spain; 3 Public Dental Health Service, Arnau de Vilanova Hospital, Valencia, Spain

**Keywords:** Orthodontics, Cephalometric, Clinical outcome, Lingual orthodontics, Labial orthodontics, Treatment

## Abstract

**Background:**

An evaluation is made of possible differences in treatment effects between labial and lingual fixed appliances.

**Methods:**

A comprehensive search was made of the PubMed-Medline, Cochrane Library and LILACS databases, with an additional manual search covering the period up until April 2017. There were no restrictions in terms of year of publication or language. Agreement between the authors was quantified by the Cohen kappa statistic. A random-effect model was applied to calculate weighted mean differences with 95% confidence intervals.

**Results:**

A total of 249 patients corresponding to four eligible studies were included in the systematic review. Among the six angles and distances entered in the meta-analysis, a tendency was observed in lingual appliances to increase the interincisal angle (95% CI −0.80-8.99; *p* = 0.101) and reduce the angle between the major axis of upper central incisor and the sellar-nasion plane - though statistical significance was not reached (95% CI −5.75-0.32; *p* = 0.079).

**Conclusion:**

The results obtained indicate that treatment with lingual appliances favors incisor tipping by exerting lingual crown torque, but there are no differences in cephalometric values between labial and lingual fixed appliances. Because of the small number of included studies, the results of this meta-analysis should be interpreted with caution. Future research should focus on the generation of a consensus document allowing selection of the type of orthodontic approach not only conditioned to the esthetic requirements of the patient but also considering the characteristics of the malocclusion. On the other hand, standardized international guidelines are lacking; the measurements of angles and distances therefore have to be unified with a view to future investigations.

## Background

Lingual orthodontics were introduced over three decades ago [1], and in recent years the demand and thus provision of lingual orthodontic treatments have increased among patients seeking improved esthetic effects [2]. A number of recent studies [3, 4] have attempted to establish the advantages and inconveniences of lingual orthodontic appliances versus labial appliances.

Cephalometric analysis is used to study the craniofacial structures of the patient, and its results have an impact on treatment planning. Cephalometry is not a direct method for diagnosing the patient conditions, yet it offers details on the craniofacial structures of the patient and thus yields diagnostic information that is helpful in defining the orthodontic treatment strategy [5]. With the advent of the computer age and our ever changing technological environment, digital imaging systems are becoming increasingly more popular than conventional film-based radiography. It is now possible to perform cephalometric tracing both through the use of digitizers and directly on screen-displayed digital images [6]. Cephalometric analysis is widely used to evaluate the changes occurring after treatment with fixed appliances [7–9], with the use of Herbst, Twin-Block [10] or Frankel systems [11], or to determine the cephalometric standards in a specific population [12]. Cephalometric study is important for ensuring correct stability, for example through control of the position of the lower incisors with respect to point A and the pogonion (A-Po) and mandibular lines, and it must be taken into account that the movement of the lower incisors towards the A-Po line should not exceed ±2 mm from the original position [13, 14]. Case reports in the literature have shown generally favorable clinical and cephalometric changes in patients treated with lingual appliances [8, 15–18]. There have been several biomechanical and in vitro studies [19–22] and case reports [23] related to lingual appliances, but only a few clinical studies [7–9] have compared their clinical outcome with that of labial appliances.

Apart from the undeniable esthetic advantages of lingual versus labial appliances, other biomechanical advantages have been described referred particularly to expansion, open bite or mass retraction [24], torque, inclination and rotation of the teeth [25, 26]. However, little evidence has been produced by clinical studies in this field, and a systematic review is therefore needed with the purpose of finding answers. Some systematic reviews and meta-analyses have been published comparing the adverse effects of labial and lingual fixed appliances [27, 28]. However, to our knowledge, this is the first review to evaluate possible differences in treatment effects between the two techniques from the radiographic perspective, based on changes in cephalometric parameters.

## Methods

### Protocol and registration

This meta-analysis was based on the guidelines provided by the Preferred Reporting Items for Systematic Reviews and Meta-analysis (PRISMA) statement [29] and the Cochrane Handbook for Systematic Reviews of Interventions (version 5.1.0) [30]. The protocol was not registered.

### PICO question

On the basis of the acronym “PICO” (Patient, Intervention, Comparison, Outcome), the question that guided this review was: Are there differences in treatment effects between labial and lingual fixed appliances?

### Search strategy

We searched the PubMed-Medline, Cochrane Library and LILACS databases, covering the period up until April 2017. The specific search strategies are shown in Table 1. Two authors (F.A.A. and J.A.A.) read the titles and abstracts of all the studies without blinding of the names of the authors, names of the journals, or publication dates. They in turn reviewed the full text articles of the potentially relevant titles and abstracts against the inclusion criteria. The search was completed with a review of the references cited in the selected articles in order to identify additional studies not found in the initial search. In addition, a manual search (likewise up until April 2017) was made of the following journals: American Journal of Orthodontics and Dentofacial Orthopedics, The Angle Orthodontist, European Journal of Orthodontics and Orthodontics & Craniofacial Research.

**Table 1:** Electronic databases searched and search strategies used in the meta-analysis (up to April 2017)

Database	Search strategy used	Hits
MEDLINE searched via Pubmed (www.ncbi.nlm.nih.gov/pubmed)	(orthodontics [mesh] OR orthodontic) AND (“labial” OR “labial orthodontics” OR “labial treatment” OR “labial bracket” OR “labial cephalometric” OR “buccal” OR “buccal orthodontics” OR “buccal treatment” OR “buccal bracket” OR “buccal cephalometric” AND (“lingual” OR “lingual orthodontics” OR “lingual treatment” OR “lingual bracket” OR “lingual cephalometric”	2642
Cochrane Central Register of Controlled Trials searched via the Cochrane Library (www.thecochranelibrary.com)	(orthodontics OR orthodontic) AND (“labial” OR “labial orthodontics” OR “labial treatment” OR “labial bracket” OR “labial cephalometric” OR “buccal” OR “buccal orthodontics” OR “buccal treatment” OR “buccal bracket” OR “buccal cephalometric”) AND (“lingual” OR “lingual orthodontics” OR “lingual treatment” OR “lingual bracket” OR “lingual cephalometric”	52
LILACS (http://lilacs.bvsalud.org/es)	(orthodontics OR orthodontic OR labial OR buccal OR lingual) AND (bracket OR cephalometric OR treatment)	191
Total		2885

### Study screening criteria

A specific protocol was conducted in advance. The following inclusion criteria were established:

1. Studies comparing changes in the cephalometric values of patients with malocclusions requiring orthodontic treatment, in which one group of subjects received treatment with the lingual technique while the other group received treatment with the buccal technique.
2. Randomized controlled trials (RCTs), prospective controlled trials (CCTs) and retrospective studies.

Patients with previous phase 1 treatment or surgical treatment, patients with continuing dental growth and in vitro or animal studies were excluded. Any obscure or missing data were obtained by contacting the authors. The electronic search included all articles, with no restrictions in terms of year of publication or language. In those cases involving more than one publication with the same group of patients and the same follow-up period, we only included the study that came closest to the objectives of the present review or which comprised the largest sample. A translation was arranged for one article in Chinese [31]. All the articles selected in the electronic and manual searches were evaluated independently by the first and second authors of the present study, in accordance with the established inclusion criteria. Any disagreements between the authors were resolved by consensus or by consulting the last author of the present study, and the level of agreement between the two reviewing authors was assessed based on the Cohen kappa statistic.

### Assessment of risk of bias of the studies

Two of the authors (F.A.A. and J.A.A.) independently assessed the risk of bias of the included randomized controlled trials using the Cochrane Collaboration tool for assessing risk of bias of RCTs [30]. The criteria included: sequence generation; allocation sequence concealment; blinding; incomplete outcome data; selective outcome reporting and other sources of bias. RCTs with inadequate random sequence generation, allocation concealment or reporting bias were considered as studies with a high risk of bias. When sufficient information was not provided on these different domains of bias to allow definite judgment, we considered that the risk of bias was unclear. In contrast, when a study was free of these biases, we considered the risk of bias to be low. We excluded studies from our meta-analysis if they had a high risk of bias [30].

The risk of bias of the included non-randomized studies was independently assessed by the same authors using the Newcastle-Ottawa Scale (NOS) [32]. The NOS is a quality assessment tool for non-randomized studies. It uses a “star system” based on three major perspectives: the selection of the study groups (0–4 stars); the comparability of the groups by controlling for first and second most relevant factors (0–2 stars); and the ascertainment of outcome of interest (0–3 stars). A total score of three or less was regarded as indicative of poor quality, 4–6 was regarded as moderate quality, and 7–9 was regarded as high quality. We excluded studies from our meta-analysis if they had poor quality. Any disagreements between the authors were resolved by consensus or by consulting the last author of the present study, and the level of agreement between the two reviewing authors was assessed based on the Cohen kappa statistic.

### Statistical analysis

The present meta-analysis is based on the inverse-variance method of DerSimonian and Laird [33], with the weighted mean difference (WMD) being taken as the measure of effect. The estimates for a random effects model were obtained. The WMD estimates are accompanied by the corresponding 95% confidence interval (95%CI) and *p*-value of the null effect contrast of the factor “type of orthodontic treatment” (WMD = 0) for solution of the meta-analysis. Graphic representation using Forest plots was made for WMD. Due to the diversity of the analyzed measurements, and taking into account that the quantitative analysis was made independently for each of the measurements, we obtained up to six independent meta-analyses. The Bessel correction was used to integrate a part of the meta-analysis [9]:

$$ {\displaystyle \begin{array}{l}\overline{X}=\frac{\sum_{i=1}^k{n}_i{\overline{x}}_l}{\sum_{i=1}^k{n}_i}\\ {} Sp=\sqrt{\frac{\sum_{i=1}^k{S}_i^2\left({n}_i-1\right)}{\sum_{i=1}^k{n}_i-k}}\end{array}} $$

where $\overline{X}$ is the average of the individual “means” ${\overline{X}}_{\mathrm{i}}$; n_i_ is the size of the *k* studies or subgroups, and S_i_ is the standard deviation of each study or subgroup.

The statistical analysis was performed using the SPSS version 17.0 statistical package for Microsoft Windows (SPSS, Chicago, IL, USA) and R version 3.0.2 (R Foundation for Statistical Computing, Vienna, Austria) [34].

### Assessment of heterogeneity and publication bias

The *I* ^*2*^ statistic describing the percentage of the total variation across studies that is attributable to heterogeneity rather than chance was used to assess between-study heterogeneity. The *I* ^*2*^ statistic is calculated as follows: 100% × (Q-df)/Q, where “Q” is Cochran’s heterogeneity statistic and “df” represents the degrees of freedom. Conventionally, I^2^ values of 25%, 50%, and 75% indicate low, moderate, and great heterogeneity, respectively [35]. Forest plots involve a weighted compilation of all the effect sizes reported by each study, and also provide an indication of heterogeneity between studies. Potential publication bias was evaluated by visual inspection of the funnel plots and was quantitatively assessed using Begg’s rank correlation test. Funnel plots and Begg’s test were chosen to detect publication bias if the number of included studies exceeded ten [30, 36].

## Results

### Selection of the included studies

**Fig. 1:**
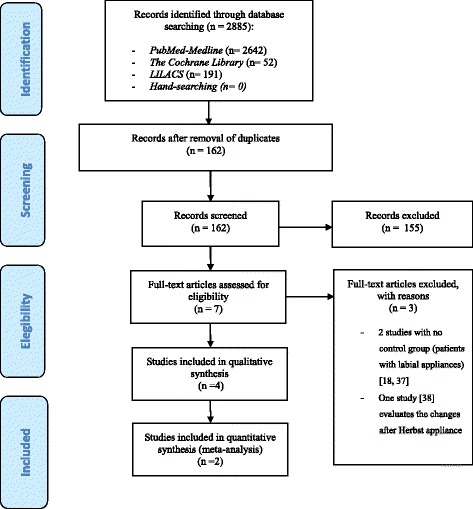
Prisma® flow diagram of the search processes and results

The electronic search procedures and excluded articles (along with the reason for exclusion) are described in Fig. 1. A total of four articles [7–9, 31] were finally included in the systematic review, and a meta-analysis was made of two studies [8, 9]. The work of Soldanova et al. [7] was excluded because the angles and measurements made could not be integrated within the quantitative analysis. The study by Wang et al. [31] was not included in the meta-analysis, since according to the Cochrane Handbook for Systematic Reviews of Interventions version 5.1.0 [30], studies with a high risk of bias are not considered for quantitative analysis. The two studies included in the meta-analysis totaled 85 patients subjected to labial orthodontic treatment and 84 patients treated with lingual brackets. The demographic data (number of patients, mean age and sex), the type of study, and the information relating to the brackets system used in each study are summarized in Table 2. The inter-rater reliability based on the kappa statistic was 0.91.

**Table 2:** Demographic data, study design characteristics and type of brackets used in the publications included in the systematic review

Study authors and design		Demographic data			Type of brackets	
Authors	Study design	No. patients (La/Li)	Sex (M/F)	Age labial/lingual (years)	Labial	Lingual
Gorman and Smith 1991 [9]	CCT	120 (60/60)	NA	NA	NA	Ormco Corp., Glendora, Calif.
Soldanova et al. 2012 [7]	CCT	50 (25/25)	11 M/39F	18–54/19–46	Roth (Dentaurum, Ispringen, Germany)	Forestadent, St Louis, Missouri, USA.
Wang et al. 2014 [31]	RCT	30 (15/15)	12 M/18F	NA	NA	NA
Deguchi et al. 2015 [8]	R	49 (25/24)	9 M/40F	24.2 ± 4.1/26.4 ± 4.7	NA	STb lingual bracket (Ormco, Orange, Calif)

*RCT* randomized controlled trial, *CCT* controlled clinical trial, *R* retrospective, *La/Li* number of patients treated with labial/lingual system, *M* male, *F* female, *NA* not available

### Description of the included studies

Four studies were included in this systematic review, and the angles and distances measured in each study are summarized in Table 3:

- *Gorman and Smith* [9]: The global 120 patients were treated in three dental clinics, and for the statistical analysis were divided into six groups according to the orthodontic technique used (labial or lingual) and the dental clinic where treatment was provided. All measurements were made before and after treatment. No statistically significant differences in treatment results between labial and lingual appliances were recorded. Significant differences in results were found only when the cases were grouped with respect to practitioner or extraction pattern, rather than the type of appliance used.
- *Soldanova* et al. [7]: This study measured the position of the lower incisor with respect to the line formed by point A and the pogonion (A-Po) and mandibular lines (ML), and the position of the lower incisor apex. For this measurement, points C and B were constructed. Point B was set as the point of intersection of the connecting line between the A-Po point and ML, and point C as the intersection of the lower incisor axis and ML. All measurements were made before and after treatment. These authors obtained significant results (*p* = 0.032) referred to the position of the incisors with respect to the A-Po line.
- *Wang* et al. [31]: X-ray cephalometric measurements were taken using Steiner and Tweed cephalometric analyses, with pre-treatment versus post-treatment comparison of the results. The angles and distances measured in the Steiner analysis were: SNA, SNB, ANB, SND, 1-NA angle, 1-NA distance, 1-NB angle, 1-NB distance, Po-NB distance, I-I (interincisal angle), OP-SN, Go-Gn-SN, SL and SE. The measurements made in the Tweed analysis were: FMA (Frankfurt-mandibular plane), FM1A (Frankfurt-lower incisor) and IMPA (lower incisor-mandibular plane). There were no statistically significant differences (*p* > 0.05) between groups in terms of each measured value recorded by Steiner and Tweed analysis after and before orthodontic treatment.
- *Deguchi* et al. [8]: All measurements were made before and after treatment. These authors recorded statistically significant results (*p* < 0.05) in the measurement of IIA and SN-U1. They also obtained significant results in the measurement of OJ and PTM-U6[PP].

**Table 3:** Angles and distances measured in the studies included in the systematic review

	Gorman and Smith [9]	Soldanova et al. [7]	Wang et al. [31]	Deguchi et al. [8]
Angles	U1-SN^a^/L1-MP^a^/U1-L1^a^/MP-OP/SN-MP^a^/SNB^a^/N-S-Gn	Position of the incisors relative to mandibular line (ML)	SNA/SNB/ANB/SND/1-NA/1-NB/IIA/OP-SN/GoGn-SN/FMA/FM1A/IMPA	SNA/SNB^a^/ANB/SN-U1^a^/IIA^a^/MP-SN^a^/L1-Mp/Occl-Pl
Distances (mm)	ULi-SN0/ULcr-SN LPi-MP/LIcr-MP/S-Gn/Me-N^a^/Ans-Me S-Go	Position of the incisors relative to A – Po Distance of incisor apex CB – C ′ B^b^	1-NA/1-NB/Po-NB/SL/SE	S-N/N-Me^a^/OJ/OB PP-U1/PP-U6/PTM-U6[PP]/MP-L1^a^/MP-L6/L6-B[MP]

See abbreviations

^a^Angles and/or distances included in the meta-analysis

^b^Points C and B were constructed. Point B was defined as the point of intersection of the connecting line between the A-Po point and ML, and point C as the intersection of the lower incisor axis and ML

### Assessment of risk of bias of the studies

The only RCT included in the systematic review was considered as having high risk of bias for inadequate allocation sequence, concealment and selective outcome reporting. Following the norms of the Cochrane Handbook for Systematic Reviews of Interventions (version 5.1.0) [30], we excluded this study [31] from our meta-analysis because of the high risk of bias.

With regard to the three non-randomized studies [7–9], two publications [7, 8] were considered to be of high quality, with an NOS score of 7, while one study [9] was considered to be of moderate quality, with an NOS score of 5 (Table 4). The agreement between the reviewers for risk of bias assessment, based on the kappa statistic, was 0.92.

**Table 4:** Newcastle-Ottawa Scale (NOS) summary assessment of risk of bias for non-randomized studies included in the systematic review

Quality criteria	Selection (4)				Comparability (2)	Exposure (3)			Total (9)
	Is case definition adequate? (1)	Representativeness of the cases (1)	Selection of controls (1)	Definition of controls (1)	Comparability on basis of design or analysis (2)	Ascertainment of exposure (1)	Same method of ascertainment for cases and controls (1)	Non-response rate (1)	
Gorman and Smith 1991 [9]	●	●	○	●	○ ○	●	●	○	**5**
Soldanova et al. 2012 [7]	●	●	○	●	● ●	●	●	○	**7**
Deguchi et al. 2015 [8]	●	●	○	●	● ●	●	●	○	**7**

### Assessment of heterogeneity and publication bias

**Fig. 2:**
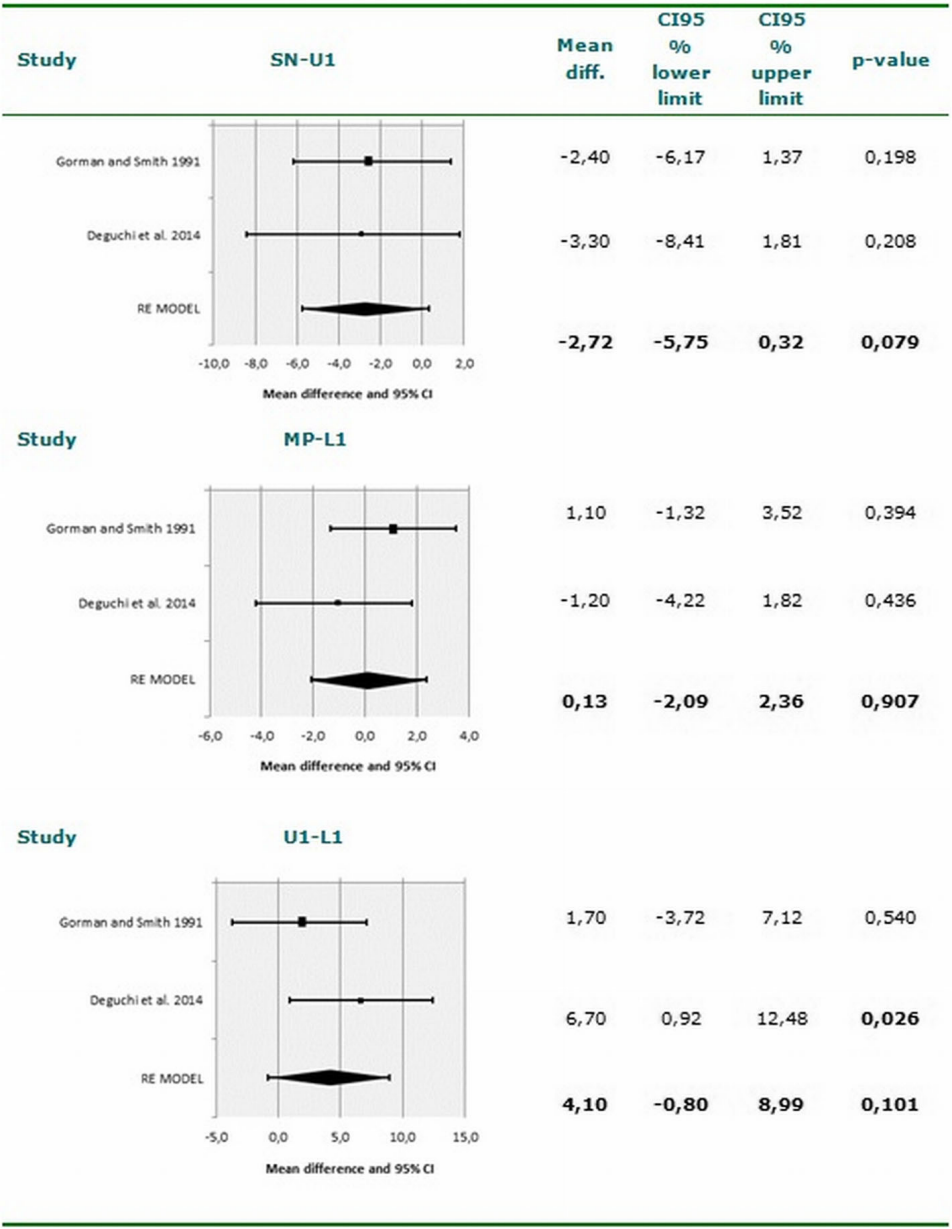
Meta-analysis corresponding to the cephalometric changes (SN-U1, MP-L1 and U1-L1). Forest plot for the mean difference including the number of source studies, effect sizes with 95% confidence intervals, and statistical significance

**Fig. 3:**
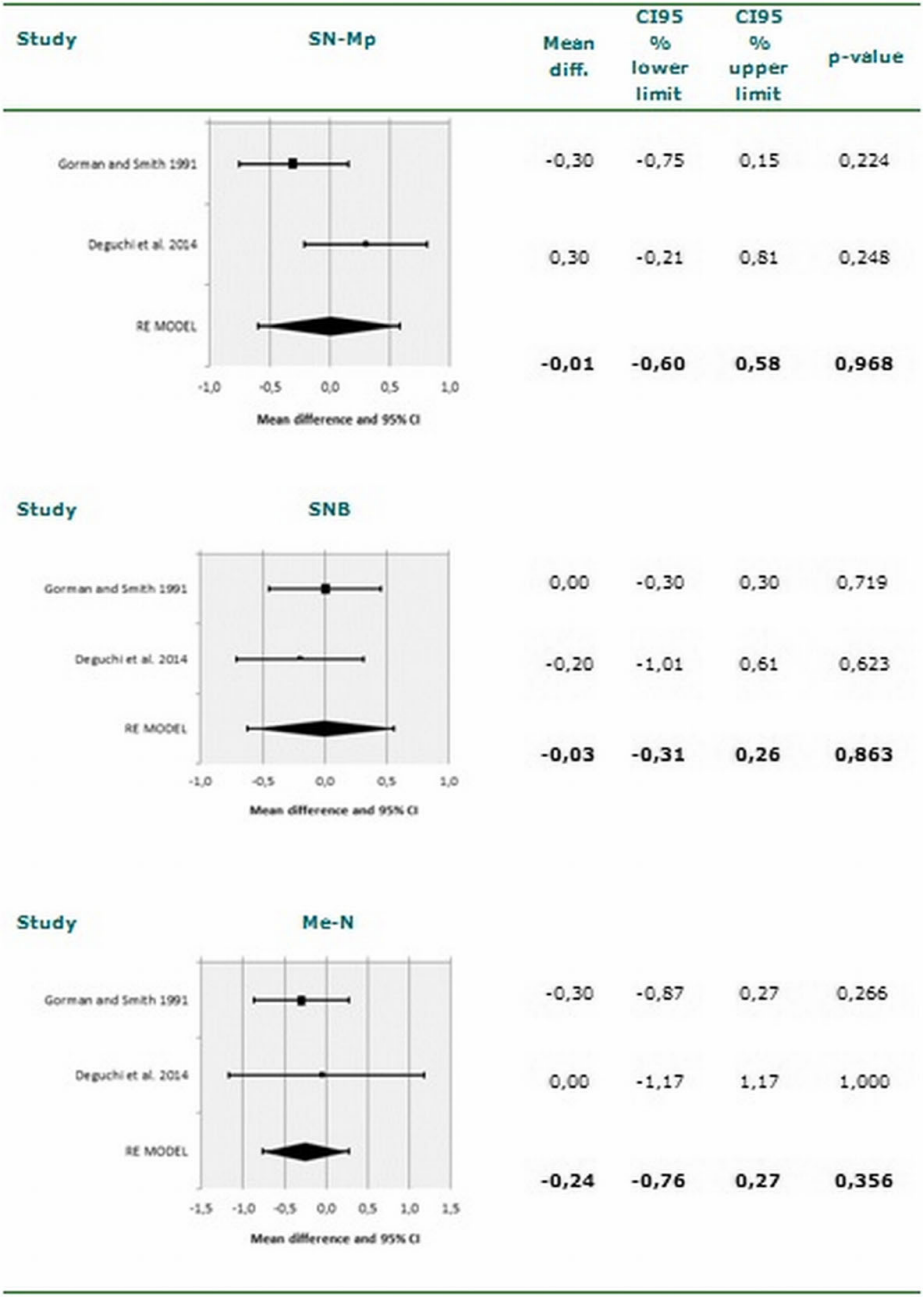
Meta-analysis corresponding to the cephalometric changes (SN-Mp, SNB and Me-N). Forest plot for the mean difference including the number of source studies, effect sizes with 95% confidence intervals, and statistical significance

The analyses of heterogeneity were based on visual inspection of the forest plots, the Q statistic and the corresponding *p*-value, and the I^2^ index. The corresponding forest plots in these analyses (Figs. 2 and 3) indicate important homogeneity across the considered studies. We recorded values of I^2^ = 0%, *p* = 0.781; I^2^ = 26.3%, *p* = 0.244; I^2^ = 34.6%, *p* = 0.216; I^2^ = 66.9%, *p* = 0.082; I^2^ = 0%, *p* = 0.649; and I^2^ = 0%, *p* = 0.651 referred to the measurements SN-U1, Mp-L1, U1-L1/IIA, SN-MP, SNB and Me-N, respectively. These values indicate the absence of heterogeneity among the included studies. None of the analyzed variables yielded I^2^ > 75% indicative of high heterogeneity. However, moderate heterogeneity was observed in the measure of the SN-MP angle, with I^2^ = 66.9%. Funnel plots and Begg’s rank correlation test were chosen to detect publication bias if the number of included studies exceeded ten*.* In the present meta-analysis, the number of studies was no greater than ten; funnel plots and Begg’s rank correlation test were therefore not performed [30, 36].

### Meta-analysis

The two articles included in the meta-analysis summed a total of 85 patients subjected to labial orthodontic treatment and 84 patients treated with lingual brackets. Meta-analysis was performed of five angles (SN-U1, Mp-L1, U1-L1/IIA, SN-MP and SNB) and a linear measurement (Me-N). A nonsignificant tendency was observed in lingual orthodontics to increase the U1-L1/IIA angle (95% CI −0.80-8.99; *p* = 0.101) and reduce the SN-U1 angle (95% CI −5.75-0.32; *p* = 0.079). The results of the meta-analysis for each measure are shown in Figs. 2 and 3. The effect of the studied variables upon the lingual appliance is summarized in Table 5. The statistical power was calculated to identify a mean effect size (Cohen d = 0.5) as being statistically significant, with a 95% confidence level. The statistical power of the study was 90%.

**Table 5:** Effect of the studied variables upon the lingual appliance

	U1-SN	L1-MP	U1-L1/IIA	SN-MP	SNB	Me-N
Fulmer and Kuftinec 1989^a^ [40]	NA	NA	+	NA	NA	NA
Gorman and Smith 1991 [9]	–	–	–	–	–	–
Gimenez et al. 2010^a^ [18]	NA	NA	NA	NA	NA	NA
Soldanova et al. 2012 [7]	NA	NA	NA	NA	NA	NA
Wang et al. 2014 [31]	NA	NA	NA	NA	NA	NA
Deguchi et al. 2015 [8]	+	–	+	–	–	–
Bock et al. 2016^a^ [44]	NA	NA	NA	NA	–	NA

*NA* not available; +: Significant difference (*p* < 0.05); −: Nonsignificant (*p* > 0.05)

^a^Studies excluded during the search process. See Fig. 1 for reasons for exclusion

## Discussion

The present study offers a systematic review and exhaustive meta-analysis of the scientific literature, with the aim of answering the following question: Are there differences in treatment effects between labial and lingual fixed appliances? Cephalometric analysis confirmed that there was minimal change in the mandibular plane angle in the lingual group, though there was a slight tendency for the maxillary molars to extrude [8]. Although from the cephalometric perspective the findings were similar in both groups, lingual orthodontics showed a tendency to increase the U1-L1/IIA angle and reduce the SN-U1 angle. Although there are some limitations, the results obtained offer useful clinical information that may serve as the basis for future investigations.

Some studies [20–22] have compared the lingual orthodontic technique versus the labial technique. Most publications found to date have been in vitro studies comparing overall retraction between the two orthodontic techniques [20], frictional resistance between the bracket lingual brackets and the labial brackets [21], or the torque generated by the two techniques [22]. Some authors [37–39] have observed changes in the cephalometric measurements before and after labial orthodontic treatment, though few studies [7–9, 31] have compared cephalometric parameters between the labial and lingual techniques used. One study [40] analyzed four clinical situations (bite opening, incisor inclinations and torque control, incisor intrusion and soft-tissue profile) in 36 patients subjected to lingual orthodontic treatment. These authors found lingual appliances to apparently cause intrusion of incisors and extrusion of molars, resulting in clockwise mandibular rotation. Another study [41] in 34 patients involved measurement of the angulation between the mandibular plane and the long axis of the mandibular central incisor, the distance from infradental level to the “D” point (bone height), and the distance from the incisal edge of the mandibular central incisor to the “D” point. The results obtained showed 57.6% of the cases to present an increase in labial alveolar bone height, while 30.3% exhibited a decrease in value, and 12.1% presented no change with the decrease in angulation between the long axis of the lower incisor and the mandibular plane (Go-Gn). The group presenting increases showed a significant prolongation of the distance “incisal edge to D point”, whereas this dimension decreased significantly in the rest of the cases.

Although it is more difficult to control incisor torque with the lingual orthodontic technique, a study [42] has found retraction of the upper and lower incisors to be greater with lingual orthodontic treatment. A three-dimensional finite element study [19] found lingual crown tipping to be more exaggerated with lingual appliances than with labial appliances. These results are consistent with those obtained in our systematic review, since lingual appliances tended to tip incisors by exerting lingual crown torque to a greater extent than labial appliances. However, once lingual crown tipping occurs, it is more difficult to correct with lingual orthodontics than with labial orthodontics [19]. A study based on a mathematical model found the application of an intrusion force using lingual brackets to create clockwise rotation and lingual crown movement, while the application of an extrusion force using lingual brackets created counterclockwise rotation and labial crown movement. These findings have important clinical implications, since in malocclusions characterized by incisor retroclination (e.g., Class II, Division 2), intrusive force applied on a retroclined incisor using lingual brackets could aggravate the initial tooth position, making the tooth more retroclined. Clockwise rotation develops, aggravating the initial retroclination through labial root movement. The opposite occurs when extrusive force is applied with lingual brackets. An extrusive force on a retroclined tooth creates counterclockwise rotation, which may improve the inclination of the incisor [43]. One study compared the dental-skeletal changes in 18 patients with lingual appliances versus 18 patients with labial appliances. The effectiveness of the Herbst appliance was evaluated in both groups by means of cephalometric analysis performed before and after treatment – the two techniques being found to yield similar results [44].

With a prevalence of malocclusion of 45.6% among males between 18 and 21 years of age, it is easy to intuit that almost one-half of all evaluated patients will require orthodontic treatment [45]. Incisor teeth crowding and misalignment of lower incisors are the most common types of malocclusions. In this systematic review, four publications [7–9, 31] evaluated the cephalometric changes associated to the two orthodontic techniques, with very similar results referred to both orthodontic approaches. However, in the clinical setting, the practical implication may be that the tendency observed in lingual orthodontics to tip the incisor crown lingual and reduce the inter-incisor angle is favorable in certain types of malocclusion such as biprotrusion cases. On the other hand, what proves advantageous in cases of biprotrusion may prove inconvenient in situations requiring increased inclination of the upper incisors, as in Class II Division 2 malocclusions. This may be due to the biomechanical differences between the two types of orthodontic approaches – lingual brackets operating closer to the center of resistance. Furthermore, there is greater torque control, since the vertical position of the point of application of the force is taken into account. The point of application in the lingual arches is more gingival than in the labial arches, thereby allowing greater anterior tooth torque control. The lesser interbracket distance in the case of lingual orthodontics may be another implicated factor [24].

Although the results of our study must be viewed with caution, they could represent a starting point for future research leading to the generation of a consensus document allowing selection of the type of orthodontic approach not only conditioned to the esthetic requirements of the patient but also considering the characteristics of the malocclusion. On analyzing the parameters measured by the different studies, only some of them appear to show some coincidence, for example referred to SNA and ANB [8, 31], SNB [8, 9, 31], and the angles U1-SN, L1-MP and SN-MP [8, 9]. In contrast, in relation to the measured distances, none of the four articles cited in this study appear to coincide; the resulting arbitrariness therefore precludes comparison. This is due to the great variety of existing cephalometric analyses attempting to assess one same parameter but in different ways. As an example, assessment of the maxillary skeletal anomalies in the cephalometric analysis published by Steiner is based on measurement of the SNA angle, while in the analysis of Ricketts it is based on maxillary depth and facial convexity. In the same way that soft tissue analysis has been standardized in orthognathic surgery based on the analysis of Arnett and Bergman [46, 47], international consensus would be needed referred to cephalometric studies in orthodontics, in order to prevent some articles from being neglected due to the impossibility of establishing comparisons with other publications – all because each investigator indistinctly uses one analytical approach or other.

### Strengths and limitations of study

Our study has several strengths. A particular strength is that in order to minimize publication bias, we performed a systematic search of different international medical databases. Moreover, no language limitation or publication date was set in order to ensure inclusion of as many data as possible from appropriate studies. Two reviewers independently chose, extracted, and evaluated data quality in order to reduce bias and transcription errors. In addition, the statistical power of the study was 90%. To our knowledge, this is the first systematic review and meta-analysis to evaluate possible differences in treatment effects between labial and lingual fixed appliances. However, our systematic review also has certain limitations. A first limitation is the small number of articles available for review, which may cause penalization by a degree of type β error. Secondly, since fewer than 10 studies were included, funnel plots and Begg’s rank correlation test were not performed [30, 36]. Lastly, we must mention the fact that the publications included in the meta-analysis were non-randomized studies, which are rated as having a lower level of evidence than randomized controlled trials (RCTs).

## Conclusions

The present systematic review found no statistically significant cephalometric differences between the lingual and labial orthodontic techniques. However, there was a tendency to increase the interincisal angle and reduce the angle between the major axis of the upper central incisor and the sellar-nasion plane. These findings indicate that treatment with lingual appliances favors incisor tipping by exerting lingual crown torque. However, because of the small number of included studies, the results of this meta-analysis should be interpreted with caution. Future research is advisable, leading to the generation of a consensus document allowing selection of the type of orthodontic approach not only conditioned to the esthetic requirements of the patient but also considering the characteristics of the malocclusion. On the other hand, standardized international guidelines are lacking; the measurements of angles and distances therefore have to be unified with a view to future investigations.

## Abbreviations

ANB: Difference between angles SNA and SNB
Ans-Me: Distance between the anterior nasal spine and chin
CCTs: Prospective controlled clinical trials
FM1A: Frankfurt-lower incisor
FMA: Frankfurt-mandibular plane
Go-Gn: Angulation between the long axis of the lower incisor and the mandibular plane
IIA: Interincisal angle
IMPA: Lower incisor-mandibular plane
L1-Mp: Angle between the major axis of the lower central incisor and mandibular plane
L1-MP: Angle between the major axis of the lower central incisor and the mandibular plane
L6-B[MP]: Horizontal distance between point B and the upper first molar cuspid over the palatine plane
LIcr-MP: Perpendicular distance between the lower incisor resistance center and mandibular plane
LPi-MP: Perpendicular distance between the lower incisal margin and mandibular plane
Me-N: Distance between the chin and nasion
ML: Mandibular lines
MP-L1: Distance between the mandibular incisal edge to mandibular plane
MP-L6: Distance between the upper first molar cuspid and mandibular plane
MP-OP: Angle between occlusal plane and mandibular plane
MP-SN: Angle between the mandibular plane and sellar-nasion plane
N-Me: Distance between the nasion and chin (anterior facial height)
N-S-Gn: Angle between the facial axis and sellar-nasion plane
OB: Vertical distance between the upper and lower incisal margin
Occl-Pl: Angle between the occlusal plane and sellar-nasion plane
OJ: Horizontal distance between the upper and lower incisal margin
PP-U1: Distance between the upper incisal margin and palatine plane
PP-U6: Distance between the upper first molar cuspid and palatine plane
PTM-U6[PP]: Horizontal distance between the pterygomaxillary fossa and upper first molar cuspid over palatine plane
RCTs: Randomized controlled trials
S-Gn: Distance between the sellar plane and gnathion
S-Go: Distance between the sellar plane and gonion
S-N: Distance between the sellar plane and nasion
SNA: Angle between the nasion plane-point A and sellar-nasion plane
SNB: Angle between the nasion plane-point B and sellar-nasion plane
SN-MP: Angle between the mandibular plane and the sellar-nasion plane
SN-U1: Angle between the major axis of the upper central incisor and sellar-nasion plane
U1-L1: Interincisal angle
U1-SN: Angle between the major axis of the upper central incisor and sellar-nasion plane
ULcr-SN: Perpendicular distance between the upper incisor resistance center and sellar-nasion plane
ULi-SN: Perpendicular distance between the upper incisal margin and sellar-nasion plane
